# Re-Thinking Anxiety: Using Inoculation Messages to Reduce and Reinterpret Public Speaking Fears

**DOI:** 10.1371/journal.pone.0169972

**Published:** 2017-01-26

**Authors:** Ben Jackson, Josh Compton, Ashleigh L. Thornton, James A. Dimmock

**Affiliations:** 1 School of Sport Science, Exercise and Health, The University of Western Australia, Perth, Australia; 2 Institute for Writing and Rhetoric, Dartmouth College, Hanover, New Hampshire, United States of America; IRCCS Istituto Auxologico Italiano, ITALY

## Abstract

Inoculation theory offers a framework for protecting individuals against challenges to an existing attitude, belief, or state. Despite the prevalence and damaging effects of public speaking anxiety, inoculation strategies have yet to be used to help individuals remain calm before and during public speaking. We aimed to test the effectiveness of an inoculation message for reducing the onset of public speaking anxiety, and helping presenters interpret their speech-related anxiety more positively. Participants (*M*_*age*_ = 20.14, *SD* = 2.72) received either an inoculation (*n* = 102) or control (*n* = 128) message prior to engaging a public speaking task and reported a range of anxiety-related perceptions. Accounting for personality characteristics and perceptions of task importance, and relative to control participants, those who received the inoculation message reported significantly lower pre-task anxiety, and following the task, reported that they had experienced lower somatic anxiety, and that the inoculation message had caused them to view their nerves in a less debilitating light. Inoculation messages may be an effective strategy for helping participants reframe and reduce their apprehension about public speaking, and investigating their efficacy in other stress-inducing contexts may be worthwhile.

## Introduction

Public speaking is often viewed as a highly threatening and anxiety-inducing task [1,2]. It has been reported, for example, that public speaking anxiety is the most common social fear among the general population [3], and that concerns regarding public speaking can impede one’s work, social, and educational functioning [4,5]. Indeed, at its most severe, public speaking anxiety may be classified as a form of social anxiety disorder [6,7]; the recent fifth edition of the *Diagnostic and Statistical Manual of Mental Disorders* (DSM-5 [6]), for example, includes a ‘performance only’ specifier that is restricted to anxiety regarding speaking or performing in public. In light of the prevalence of public speaking anxiety and the deleterious implications with which it is associated, the aim of this experiment was to test the effectiveness of a novel messaging strategy designed to reduce the onset of public speaking anxiety, as well as to help presenters interpret their speech-related anxiety in a more positive light.

Sustained research attention has been devoted to studying the origins of public speaking anxiety [8], and desensitization procedures, cognitive modification therapies, and/or skills training approaches have also been implemented with the goal of helping presenters overcome (or reduce) their anxiety [8,9]. For example, treatment methods that have been successful in reducing self-reported anxiety levels include stress inoculation training [10], cognitive behavioral therapy [11], confidence-raising techniques [12], video-based methods [13], attention modification programs [14], information provision and education strategies [15,16], virtual reality training [17], and hypnosis-based approaches [18].

In light of the effectiveness of these interventions, one might reasonably question whether further investigation of public speaking anxiety treatment is required. There are, however, two key considerations that encourage additional research in this area, and that provided the rationale for this investigation. First, although these treatments have largely been effective, widespread implementation may be difficult to achieve in many instances. For example, a number of these methods are relatively time- and/or labor-intensive, and require repeat attendance, trained interventionists, and/or one-to-one administration (e.g., stress inoculation training, hypnosis-based methods, curriculum-based education). Similarly, other methods are not cost-effective or well-suited to mass dissemination (e.g., virtual reality training). Accordingly, it is important to explore cost-effective, standardized methods that are suited for widespread dissemination and do not rely on the capacity/availability of a third party. Related to the notion of developing standardized treatments, it is also noteworthy that the anxiety-reducing strategies outlined in the available literature have typically not been devised using established messaging/persuasion principles (i.e., by drawing from theoretical principles that inform us about how to generate persuasive messages that can shape or sustain desired states and attitudes). Persuasion frameworks provide guidance on the development of effective message structure and content [19], and despite their potential for use in relation to public speaking preparation, these frameworks have been absent from many of the previously published studies. These considerations (i.e., an easily administered, standardized, cost-effective method, suitable for mass dissemination and not reliant on an interventionist) were foremost in informing the development of our experimental manipulation, and we sought to ensure that our approach was scaffolded by established persuasion guidelines.

In addition to the abovementioned design considerations, studies focusing on public speaking anxiety treatment have not traditionally embraced all that is known about both the structure and interpretation of performance anxiety. It is acknowledged in public speaking [5] and other settings [20] that anxiety may consist of a physiological/somatic component (e.g., trembling body/voice, muscle tension) alongside a cognitive/worry component (e.g., self-doubt, fear of one’s anxiety being visible to the audience). Despite this perspective, some of the documented attempts to alleviate public speaking anxiety have failed to differentially or explicitly account for both somatic and cognitive components within their assessment [11,14,16].

More importantly, although these studies have been successful in reducing individuals’ self-reported anxiety, it is also important to account for individuals’ *interpretations* about the effects of any residual nerves. Anxiety interpretation (or reappraisal) research within a range of social and performance situations [21–24] has stressed the importance of understanding not only the level (or *magnitude*) of a person’s anxiety, but also the way (i.e., *direction*) in which that anxiety is appraised by the focal person. It has been demonstrated that, depending on one’s mindset and perception of control, a given amount of anxiety need not be viewed as debilitative (i.e., damaging) for one’s functioning, and may in fact be perceived as facilitative (i.e., helpful) for one’s efforts [21,23,25,26]. Previous public speaking anxiety treatments have been effective in reducing, but not completely eliminating, public speaking anxiety, and in light of the reappraisal literature that highlights individuals’ ability to perceive anxiety in a favorable light, it is important that researchers devise methods that not only help with anxiety reduction, but also assist participants in reinterpreting their remaining nerves more positively.

### Inoculation Theory: A Framework for Reducing and Reframing Anxiety?

Inoculation theory is a well-established framework [27,28] for helping individuals withstand attacks or challenges to existing attitudes, beliefs, or states. Developed out of research on the effectiveness of two-sided messaging (i.e., messages in which arguments in favor of, and opposing, a source’s position are presented [29]), inoculation theory is a resistance-based model that has its roots in a medical analogy. Specifically, medical immunization works through an adaptation process that occurs following exposure to a weakened form of a virus, thus rendering the host immune to future, stronger strains of the virus. Operating at a psychological level in much the same way, it was proposed within inoculation theory that when individuals are (a) warned of an impending threat to their current position or perception, (b) provided with examples of potential forthcoming challenges to that position/perception (i.e., counterarguments), and (c) given refutations to those arguments, this may help the individual retain his/her original position should those (or other) challenges actually occur in the future. For example, in seeking to protect individuals’ anti-alcohol attitudes, inoculation message designers might first warn recipients that they may be challenged on their stance (e.g., “there may be people who try to convince you that excessive alcohol consumption isn’t so bad after all”), before then providing the recipient with examples of likely challenges (e.g., “your friends might tell you that drinking alcohol in excess is safe and fun…”), and refutational material that explains why the recipient’s position should not be altered by those challenges (e.g., “…but, there are well-documented dangers associated with excessive alcohol consumption”). Inoculation messages have been used to successfully protect a range of important perceptual variables in health (e.g., anti-smoking and -alcohol beliefs; for a comprehensive review of health inoculation strategies [30]) and physical performance [31,32] domains, among other contexts.

There is also evidence that inoculation may be used to protect against undesired future states (e.g., feelings, cognitions). For example, Richards and Banas [33] used an inoculation strategy to protect against feelings of reactance (i.e., feeling one’s freedoms are threatened) that may be stimulated through the receipt of persuasive health messages (e.g., “don’t tell me not to smoke, I’ll do what I want”). In their inoculation treatment, recipients were explicitly warned that they may be prone to experiencing reactance following the receipt of a message encouraging the avoidance of binge drinking (i.e., “you might feel that your freedom to choose how you will consume alcohol is being threatened”). Subsequently, recipients were provided with refutational information designed to allay such responses (i.e., “However, the facts about binge drinking that are reported are pretty powerful when you think about them, and the suggestions that are proposed about drinking responsibly actually make a lot of sense”).

In relation to the aims of the present study, therefore, it could be theorized that a message developed using inoculation theory principles might help ‘protect’ speakers against the onset of anxiety, and against negative interpretations regarding the effects of anxiety. In that sense, the ‘resistance’ aspect of inoculation theory, in this instance, refers to individuals’ ability to resist the onset of debilitating anxiety prior to and during a public speaking activity (i.e., retaining a calm, controlled state). An inoculation message to protect individuals in this context, therefore, would include a threat-inducing forewarning about the anxiety that public speaking may invoke (e.g., “many presenters become nervous prior to, and during, speaking in public”), material that highlights the specific concerns that individuals may face (e.g., “you may worry that everyone can see how anxious you are”), and finally, information that helps the recipient overcome, cope with, or more positively interpret those specific concerns (e.g., “don’t worry, because in fact, people will not be able to gauge your nerves very well”). Although inoculation messages have, until now, not been used with the aim of protecting individuals against debilitating anxiety experiences, they offer a standardized, theory-driven method that is suitable for mass dissemination. As a result, this method enabled us to address a number of the treatment design considerations highlighted previously. Similarly, although reappraisal research using public speaking activities [21,25] supports the notion that participants can interpret a given level of anxiety in more or less adaptive ways, little attention has been directed toward studying the utility of reappraisal methods (a) based on inoculation message principles, (b) among non-clinical samples, or (c) when performing a ‘real-world’ activity—in front of a relatively large audience—that participants have been aware of (and able to prepare for) over a period of weeks (rather than a brief preparation period ahead of a laboratory-based task).

The aim of this experiment was to assess the effect of an inoculation message, relative to a neutral control message, on individuals’ anxiety-related perceptions immediately prior to, and during, a public speaking activity. As such, our aim was not to test the efficacy of an inoculation message in improving recipients’ public speaking *performance* per se; rather, we sought to determine whether an inoculation message—delivered prior to a public speaking engagement—may facilitate recipients’ emotions and interpretations relating to the speech. Prior to the task, we measured individuals’ social anxiety (i.e., their fear of negative audience evaluation), general anxiety about the upcoming speech, and their self-efficacy (i.e., their confidence in their ability regarding their speech). We measured self-efficacy alongside anxiety perceptions due to previous public speaking work that has demonstrated an inverse association between anxiety and self-efficacy, and has reported an efficacy-enhancing effect of anxiety reduction or reappraisal treatments [12,21]. Following the task, participants reported the level of worry (cognitive anxiety) and somatic anxiety they had experienced during the task, their interpretations of their anxiety, and the self-talk that they employed during the activity. We measured anxiety magnitude and interpretations in light of the anxiety-focused nature of the message, and included assessment of self-talk as evidence indicates that changing participants’ self-statements is an important part of an effective public speaking anxiety-reduction treatment [13]. In line with the findings for previous public speaking anxiety treatments [9], and the effectiveness of inoculation messages for protecting recipients’ perceptions [30], we hypothesized that those who received an inoculation message—relative to an information-only control message—would report lower anxiety levels, more facilitative (as opposed to debilitative) interpretations of anxiety, alongside greater pre-task self-efficacy as well as more positive and less negative self-talk.

## Method

### Participants and Procedure

Upon receiving ethical approval to conduct the study from The University of Western Australia Human Research Ethics Committee (RA/4/1/6754), participants were recruited from an undergraduate kinesiology class at the lead author’s institution, and participation was voluntary in return for class credit. Data collection took place over two years; all members of the 2014 and 2015 cohorts were invited to participate in the study. The final sample—excluding those who enrolled in the study but subsequently failed to undertake the public speaking task (*n* = 5; students who withdrew from the class during the semester)–consisted of 230 undergraduates (*M*_*age*_ = 20.14, *SD* = 2.72), who were cluster randomized (by year group) into control (*n* = 128, *M*_*age*_ = 19.92, *SD* = 2.22, 60 males, 68 females) or treatment (*n* = 102, *M*_*age*_ = 20.41, *SD* = 3.23, 50 males, 52 females) conditions. In terms of participants’ GPA, those in the control treatment had a mean GPA on entering the class of 5.13 (*SD* = 0.83, range = 2.25 to 6.75), and those in the treatment condition had a mean GPA on entering the class of 5.33 (SD = 0.97, range = 2.69 to 7.00). The sample size exceeded that which was indicated as part of an a priori sample size estimation (i.e., approximately 50–60 participants per cell) based on existing meta-analytic evidence for the magnitude of inoculation effects [34]. Students who undertook the class in 2014 were assigned to the control condition, and those who took the class in 2015 were assigned to the treatment condition. The decision to cluster randomize, rather than randomly assigning individuals within each year group, was made on the grounds that we sought to avoid the significant threat to validity associated with word-of-mouth contamination effects that would arise if those in the treatment condition were able to discuss the message with those in the control condition. In order to standardize the task across years, participants in the control and treatment conditions were assigned the exact same topics for their speeches (all speeches were based on a study that had been conducted earlier in the semester and that remained identical between 2014 and 2015), and all requirements of the activity were held constant across both years (e.g., assessor, group size, audience size, location). Prior to their involvement in the study, participants had received no formal public speaking training as part of their university degree.

In the first week of the 13-week semester, participants were informed by their lecturer that, as part of the class requirements, they would perform a 30-minute group-based speech/presentation in front of approximately 20 of their classmates. It is important to clarify that although participation in the speech was a class requirement, participation in the research procedures associated with the speech (i.e., questionnaire completion, message receipt) was voluntary. In an attempt to minimize the effects associated with being able to choose one’s group members and presentation slot (e.g., one’s anxiety being influenced by working with, or presenting in front of, one’s friends), participants were randomly assigned to a presentation group consisting of four presenters, and to a presentation slot between week 9 and 12 of the semester. Presentations were scheduled in a group-based format due to logistical reasons (i.e., fitting all presentations in during the allotted time period); however, participants were informed that their performance *as an individual* would be assessed by an instructor (who was blind to the study protocol and aims). Participants were also informed that they should split the speaking requirements evenly between all group members. As a result, although each group prepared for a 30-minute speech, individual group members spoke for approximately 8 minutes, and were made aware that they would be assessed on an individual basis regarding their performance during that period.

Having provided their written consent to participate in the research, two weeks prior to their presentation date, participants were provided with a hard copy information sheet from the lecturer that contained the control or treatment material. Participants were instructed to read the information sheet, and three days prior to their presentation, they were electronically provided with the same information sheet. The decision to provide the information sheet in advance of the presentation was taken on the basis that public speaking anxiety peaks immediately prior to an activity [35], and so we provided the material in advance of this period so as to ensure that participants were not so anxious/threatened [36] that they would be unable to attend fully to the content of the message. On the basis of their response to a screening question provided at the close of data collection, all participants verified that they had read the information provided. Immediately before and after their presentation, participants were asked to complete a series of questionnaires. It was not until data collection was completely terminated that all participants were presented with complete information about the true aims of the study (and those in the control group were provided with a copy of the inoculation treatment).

### Experimental Manipulation

Participants in the control condition received a generic one-paragraph information sheet that detailed the requirements of the activity, the nature of the assessment, the implications of their performance for their overall class grade, and wished them luck with their speech. Within the treatment condition, alongside the generic information that was presented to those in the control group, participants were provided with additional material derived in line with principles of inoculation theory (see S1 File). First, participants were provided with a forewarning regarding the anxiety that they may experience. Following this forewarning, participants in the treatment condition were presented with three counterarguments and paired (i.e., passive) refutations that targeted common preconceptions and anxiety-inducing concerns related specifically to public speaking (for support [2,16,37]). The first counterargument-refutation pairing was designed to highlight and address fears related to the ‘visibility’ of one’s anxiety, and focused specifically on reassuring individuals regarding the illusion of transparency. The second pairing focused on highlighting and alleviating concerns relating to the extent to which one would be scrutinized by the audience (i.e., the spotlight effect [38]), and the final pairing focused on drawing participants’ attention to (and minimizing concerns regarding) the detrimental effects that anxiety may have on their performance. This final pairing was designed to inform individuals that anxiety may not necessarily be damaging to their performance, and need not be interpreted in a debilitative manner. To strengthen the refutational claims, citations to empirical evidence were included (for the interested reader, these references are listed in S1 File), and the writing emphasized the relevance of the material for the participants’ speaking performance.

### Measures

#### Background variables and inoculation components: Personality traits

In order to obtain a brief assessment of personality traits, participants completed a brief version of the Big Five Inventory (BFI-10 [39]) during the first week of the semester. Participants responded to the stem, “I see myself as someone who…”, and the BFI-10 contains two items for each of the Big Five traits, namely agreeableness (e.g., “is generally trusting”), conscientiousness (e.g., “does a thorough job”), extraversion (e.g., “is outgoing, sociable”), neuroticism (e.g., “gets nervous easily”), and openness to experience (e.g., “has an active imagination”). The BFI-10 has been widely used for the purpose of brief personality assessment, and support for the validity and reliability of scores derived from the BFI-10 has been reported previously [39,40]. Commonly-used reliability estimators are not recommended as a criterion for judging the reliability of BFI-10 subscales, given that the items within each scale are designed to broadly cover the personality dimension in its entirety (rather than to assess the same facet within any given dimension [40]). Purely for information purposes, though, and given that each subscale consists of two items, we did calculate Spearman-Brown coefficients (ρ) for agreeableness (ρ = .33), conscientiousness (ρ = .47), extraversion (ρ = .71), neuroticism (ρ = .57), and openness (ρ = .39). Despite the relatively low internal consistency estimates observed for some subscales, we retained the BFI-10 scores on the basis of the conceptual argument presented above.

#### Background variables and inoculation components: Perceived threat

Consistent with previously-reported measurement procedures [31], one item was used to assess participants’ perceptions of threat relating to the speaking activity. Participants were asked to respond to the statement, “Thinking about the upcoming presentation, I view the prospect of challenges to my ability to present well as…”, using a bipolar response scale anchored at 1 (*unlikely*) and 7 (*likely*). The use of a single threat item was advantageous in order to limit overall questionnaire length, given that participants were asked to complete threat, importance, and pre-task measures immediately prior to their presentation.

#### Background variables and inoculation components: Task importance

Participants rated the importance of the speaking task using a single item (“It is important to me to do well in my presentation”), anchored at 1 (*not at all true*) and 7 (*very true*).

#### Pre-task perceptions: Social anxiety

Participants completed a revised version of an existing four-item instrument [41], which was designed to measure their anxiety regarding the way in which they would be evaluated by their classmates (i.e., the audience). Following the stem, “right at this moment in time, thinking about this presentation…”, participants responded to statements including, “I am concerned about embarrassing myself in front of the audience”, and “I am concerned that the audience will think I am a poor presenter”. Minor modifications were made to the original items in order to focus participants’ attention toward their audience, and the original response scale, anchored at 1 (*not at all*) and 5 (*extremely*), was used. The internal consistency of the measure derived from this instrument in this study was α = .87.

#### Pre-task perceptions: Task-related anxiety

Participants responded to a single item assessing the degree to which they were anxious about their presentation. Specifically, using a 5-point response scale ranging from 1 (*not at all*) to 5 (*a great deal*), participants were asked, “overall, how nervous or anxious do you feel right now about your presentation today?”

#### Pre-task perceptions: Self-efficacy

Consistent with self-efficacy scale construction guidelines [42], we assessed participants’ confidence in their ability regarding their speech with items that were devised to represent the primary tasks required of them during their presentation. Using an established response scale [43] ranging from 1 (*no confidence at all*) to 5 (*complete confidence*), participants were provided with four items (i.e., “control your nerves at all times”, “speak clearly at all times”, “maintain audience interest at all times”, and “deal well with any audience questions”) following the stem, “right at this moment in time, how confident are you in your ability to…”. The internal consistency of self-efficacy measure derived from this instrument was α = .75.

#### Retrospective assessment of in-task perceptions: Cognitive and somatic anxiety

Immediately following the task, and without having received any evaluative feedback regarding their performance, participants were asked to report the degree of cognitive and somatic anxiety they had experienced during the presentation by completing the five-item somatic anxiety subscale and a modified version of the five-item worry (i.e., cognitive anxiety) subscale from the Sport Anxiety Scale-2 (SAS-2 [44]). We recognize that the SAS-2 was developed to assess worry and somatic anxiety with respect to sporting performance contexts; however, upon inspection, the items within these subscales appeared to be either directly applicable (i.e., in the case of somatic anxiety; example items, “my body felt tense”, “my stomach felt upset”) or modifiable (i.e., worry; example revised items, “I worried that I would not present well”, “I worried that I would mess up during the presentation”) for the performance of public speaking.

Prior to deciding to use this instrument, we conducted a thorough literature search that revealed no established instrument specific to public speaking that fit our measurement criteria. As a result, although the SAS-2 was developed for a different context, we selected this instrument in light of a number of considerations. In particular, in comparison to instruments specific to public speaking anxiety that have been used previously [45–47], the benefits of using the modified SAS-2 were that it provided the opportunity to assess performance-related anxiety (a) using a brief, validated instrument, (b) in relation a specific activity (i.e., we required a situation-specific measure), (c) with respect to an activity performed alongside others (e.g., worry item, “I worried that I would let others down”), and (d) using a measure that separately assessed cognitive and somatic dimensions of anxiety. In addition, instruments prominently used to measure anxiety (e.g., Beck Anxiety Inventory [48]) or social anxiousness (e.g., Interaction Anxiousness Scale [49]) were also unsuitable given their clinical or trait-like assessment method, and their inability to be modified easily to suit public speaking situations.

In order to obtain retrospective ratings, participants were requested to respond to all statements by circling the number that best represented how they felt during their presentation, and in line with original scoring procedures, a response scale ranging from 1 (*not at all*) to 4 (*very much*) was employed. The use of retrospective assessments to assess one’s anxiety levels is well established within the public speaking literature [13,50]. Support for the reliability and structural properties of measures derived from the SAS-2 has been reported [44], and amended versions of the SAS-2 have been shown to be appropriate for use in contexts other than sport [51]. The internal consistency for measures derived from the worry (α = .91) and somatic anxiety (α = .90) subscales were acceptable in this investigation.

#### Retrospective assessment of in-task perceptions: Interpretation of anxiety

Consistent with recommendations [52] and with previously-used measures of anxiety/emotion direction [53,54], participants were asked to reflect how, on the whole, they felt their nerves/anxiety had influenced their presentation performance. Using a scale anchored at -3 (*strong negative impact*), 0 (*no impact at all*), and 3 (*strong positive impact*), participants responded to the item, “overall, how do you feel your nerves or anxiety impacted on how well you were able to present today?” As a result, a higher score on this index indicated that participants perceived their anxiety to be more facilitative for their speaking performance.

#### Retrospective assessment of in-task perceptions: Impact of message

In order to identify whether individuals’ anxiety interpretation was influenced directly by the message (i.e., information sheet) they received, participants responded to a single item (“overall, what impact did the information sheet you received have on the way you viewed your nerves or anxiety about your presentation?”), using a response scale anchored at -3 (*it made me more worried about being nervous*), 0 (*it had no impact on my interpretation of my nerves*), and 3 (*it made me less worried about being nervous*). Accordingly, a higher score on this measure indicated that participants felt the message had enabled them to interpret their nerves/anxiety more positively.

#### Retrospective assessment of in-task perceptions: Self-talk

The final instrument that participants completed following their presentation was the Self-Statements during Public Speaking (SSPS) scale [55]. The 10-item SSPS scale comprises two five-item subscales that allow researchers to retrospectively assess individuals’ positive (e.g., “I can handle everything”) and negative self-statements (e.g., “what I say will probably sound stupid”) during a public speaking task. Participants were instructed, “The statements below cover some of the things that you may have felt and thought to yourself during your presentation. Reflecting on how you felt and thought to yourself during your presentation, how much do you agree with each of the statements provided below?” Consistent with the original scoring procedures, responses were made on a 6-point scale anchored at 0 (*do not agree at all*) and 5 (*agree extremely*), and higher scores for each subscale represent greater positive/negative self-statements. Support for the structural properties, internal consistency, and test-retest reliability of measures derived for both SSPS subscales has been reported [55]. In the present study, we observed an acceptable level of internal consistency for the negative self-statements subscale (α = .82); however, the internal consistency of the positive self-statements subscale (α = .66) was marginal, and so (on conservative grounds) we excluded this subscale from further analyses.

## Results

A missing value analysis on all primary variables was conducted using IBM SPSS (Version 22.0), and indicated that the missing data (which represented less than 0.1% of the overall data file) were missing completely at random; Little’s chi-square test [56] was nonsignificant, *χ*^2^(877) = 834.87, *p* = .84, and missing data were imputed using the expectation maximization procedure. For the data file used in the analyses reported below, see S2 File.

### Preliminary Analyses

Prior to testing for between-condition differences on threat and task importance, and in light of the cluster randomization method that we employed, we sought to rule out there being any potential demographic differences between the two cohorts. A chi-square test of association for gender-by-condition revealed no significant effect, χ^2^(1) = .10, *p* = .75, indicating that the proportion of males-to-females was consistent between years (i.e., between those assigned to control versus treatment conditions), and a one-way ANOVA indicated no significant age difference between participants in the two conditions, *F*(1, 228) = 1.85, *p* = .18, η^2^_p_ = .008.

In addition to checking for demographic differences, we also tested for potential background differences in terms of participants’ personality traits and GPA between years (i.e., their GPA on entering the class). A one-way MANOVA, in which GPA and Big Five personality scores were treated as dependent variables (i.e., 6 dependent variables), and condition (i.e., control vs. treatment) was the independent factor, revealed a nonsignificant multivariate effect, *F*(6, 211) = 1.69, *p* = .12, η^2^_p_ = .05, λ = .95. As would be expected from this multivariate effect, at the univariate level there were no significant differences using a Bonferroni-adjusted alpha level for multiple comparisons (i.e., .05/6 = .008). However, in light of the significance level that we observed for the between-condition difference on extraversion, *F*(1, 216) = 4.34, *p* = .038, η^2^_p_ = .02 (*M*_*control*_ = 3.52, *SD*_*control*_ = .82; *M*_*treatment*_ = 3.28, *SD*_*treatment*_ = .94, on a 1-to-5 scale), and given the relevance of extraversion for one’s reactions to social evaluative activities [57], we adopted a conservative approach and included extraversion as a covariate when examining subsequent between-condition differences.

To examine between-condition differences on perceptions of threat and task importance (measured prior to the activity), we ran a one-way MANCOVA, with condition as the independent factor, threat and importance as dependent variables, and extraversion as a covariate. Descriptive data for these and all other variables—separated by condition—are displayed in Table 1. The analysis revealed a significant multivariate effect for condition, *F*(2, 226) = 7.87, *p* < .001, η^2^_p_ = .06, λ = .93. Using a Bonferroni-adjusted alpha criterion at the univariate level in light of multiple comparisons (i.e., .05/2 = .025), the multivariate effect was accounted for by significant differences on perceived task importance, *F*(1, 227) = 14.45, *p* < .001, η^2^_p_ = .06. Specifically, although both groups of participants endorsed strong absolute perceptions of task importance, participants in the treatment condition reported greater perceptions of importance relative to those in the control condition (see Table 1). For this reason, we entered task importance as a covariate in subsequent analyses, alongside extraversion. Univariate follow-ups revealed no significant difference for threat perceptions between conditions, *F*(1, 227) = 0.33, *p* = .57, η^2^_p_ = .001.

**Table 1:** Descriptive statistics according to condition.

	Inoculation (*n* = 102)		Control (*n* = 128)		Between-condition effect size (*d*)
	*M*	*SD*	*M*	*SD*	
*Inoculation components*					
Threat	4.40	1.38	4.53	1.18	.10
Task importance	6.40	.75	6.04	.83	.46
*Pre-task perceptions*					
Social anxiety	2.95	.93	3.21	.94	.28
Task-related anxiety	3.36	.88	3.74	.87	.43
Self-efficacy	3.01	.66	2.89	.67	.18
*Task perceptions*					
Cognitive anxiety/Worry	2.32	.75	2.56	.76	.32
Somatic anxiety	1.90	.70	2.18	.78	.38
Interpretation of anxiety	-.37	1.18	-.73	1.03	.32
Impact of message	.52	.97	-.02	.84	.60
Negative self-talk	.97	.82	1.23	.93	.30

*Note*. Threat and importance measured 1–7, where higher scores denote greater threat/importance. Social anxiety, task-related anxiety, and self-efficacy measured 1–5, where higher scores denote greater anxiety/confidence. Cognitive and somatic anxiety rated 1–4, where higher scores denote greater anxiety. Interpretation of anxiety and impact of message rated -3 to 3, where positive (negative) scores denoted a more positive (negative) interpretation of anxiety/impact of message. Negative self-talk measured 0–5, where higher scores denote greater negative self-talk. *d* column = Cohen’s *d* effect size estimate for mean between-condition comparison on each primary variable.

### Main Analyses

#### Pre-task perceptions

In light of the gender differences that have been reported previously for public speaking anxiety prevalence [5], when examining potential between-condition differences on variables measured before the speaking performance, we accounted for gender by performing a two-way MANCOVA, with gender and condition as independent factors. We included extraversion and task importance as covariates in our analyses, and entered participants’ social anxiety, task-related anxiety, and self-efficacy as dependent variables. Our analyses revealed significant multivariate main effects for condition, *F*(3, 222) = 5.01, *p* = .002, η^2^_p_ = .06, λ = .94, and gender, *F*(3, 222) = 4.63, *p* = .004, η^2^_p_ = .06, λ = .94, but no multivariate interaction effect, *F*(3, 222) = 1.29, *p* = .28, η^2^_p_ = .02, λ = .98. We followed up the condition and gender main multivariate effects using an adjusted alpha criterion at the univariate level in light of multiple comparisons (i.e., .05/3 = .017), and identified that the condition effect was accounted for by significant between-condition differences in terms of participants’ task-related anxiety, *F*(1, 224) = 13.00, *p* < .001, η^2^_p_ = .06, but not social anxiety, *F*(1, 224) = 5.05, *p* = .026, η^2^_p_ = .02, or self-efficacy, *F*(1, 224) = 0.53, *p* = .47, η^2^_p_ = .01. Relative to their counterparts in the control condition, those who received the inoculation treatment reported significantly lower anxiety regarding their speaking performance (see Table 1). The univariate significance level for participants’ social anxiety was below .05; however, this difference was not significant when accounting for the adjusted alpha criterion. In absolute terms, the mean between-condition differences on social anxiety and task-related anxiety were 0.26 and 0.38, respectively (on a 1-to-5 scoring scale).

The multivariate effect that we observed for gender—although not substantively important for the purpose of the investigation—was accounted for by significant differences between males’ and females’ social anxiety, *F*(1, 224) = 10.89, *p* = .001, η^2^_p_ = .05, task-related anxiety, *F*(1, 224) = 9.27, *p* = .003, η^2^_p_ = .04, and self-efficacy, *F*(1, 224) = 10.76, *p* = .001, η^2^_p_ = .05. In particular, prior to the speaking task, females on average reported greater social and task-related anxiety, and lower self-efficacy, compared to males. For clarity, males reported mean scores for social anxiety, task-related anxiety, and self-efficacy of 2.90 (*SD* = 0.90), 3.40 (*SD* = 0.84), and 3.08 (*SD* = .60), respectively. Females reported mean scores for social anxiety, task-related anxiety, and self-efficacy of 3.28 (*SD* = 0.94), 3.73 (*SD* = 0.91), and 2.82 (*SD* = .70), respectively.

#### Task perceptions

Our final analytic procedure focused on examining potential condition- and gender-related differences on variables relating to participants’ experiences during their presentation. To do so, we performed a two-way MANCOVA, with gender and condition as independent factors, extraversion and task importance as covariates, and participants’ (a) cognitive anxiety/worry, (b) somatic anxiety, (c) interpretation of their anxiety, (d) perception of the impact of the message on their anxiety, and (e) negative self-talk, as separate dependent variables. Analyses revealed a significant multivariate main effect for condition, *F*(5, 220) = 5.15, *p* < .001, η^2^_p_ = .10, λ = .90, alongside a nonsignificant multivariate main effect for gender, *F*(5, 220) = 2.12, *p* = .06, η^2^_p_ = .05, λ = .95, and a nonsignificant multivariate gender-by-condition interaction, *F*(5, 220) = 0.63, *p* = .68, η^2^_p_ = .01, λ = .99.

Univariate follow ups, using an adjusted alpha criterion in light of the multiple comparisons (i.e., .05/5 = .01), revealed that the main effect for condition was accounted for by differences on somatic anxiety, *F*(1, 224) = 7.37, *p* = .007, η^2^_p_ = .03, and the perceived effect of the information sheet on how participants viewed their nerves, *F*(1, 224) = 17.81, *p* = < .001, η^2^_p_ = .07. Specifically, participants who received the inoculation treatment reported reduced somatic anxiety and felt that the information sheet (i.e., message) was responsible for making them less concerned about their anxiety (i.e., it made them less worried about being nervous), relative to those in the control condition (see Table 1). In absolute terms, the mean between-condition difference on somatic anxiety was 0.28 (on a 1-to-4 scoring scale), and the mean difference for message impact—scored from -3 to 3 –was 0.54. The univariate significance level of the differences on cognitive anxiety/worry, *F*(1, 224) = 5.90, *p* = .016, η^2^_p_ = .03, and participants’ interpretation of their anxiety, *F*(1, 224) = 4.94, *p* = .027, η^2^_p_ = .02, was also below .05; however, these differences were not significant when accounting for the adjusted alpha criterion. The univariate significance level for the remaining variable in the model, namely negative self-talk, was *F*(1, 224) = 3.44, *p* = .065, η^2^_p_ = .02. Zero-order correlations between all primary variables are presented in Table 2.

**Table 2:** Aggregate-level skewness, kurtosis, and zero-order correlations for all variables (including GPA) across the entire sample.

Variable	Skew.	Kurt.	2	3	4	5	6	7	8	9	10	11
1. GPA	-.44	-.05	-.05	.09	.01	-.05	.19**	-.09	-.09	.10	.08	-.14*
2. Threat	-.37	-.01	-	.15*	.13	.11	.12	.15*	.12	.02	-.01	.06
3. Task importance	-.83	.18		-	-.07	-.01	.26***	-.06	-.08	.12	.05	-.18**
4. Social anxiety	-.04	-.55			-	.69***	-.65***	.66***	.55***	-.18**	-.16*	.54***
5. Task-related anxiety	-.17	-.52				-	-.53***	.57***	.59***	-.23***	-.22**	.39***
6. Self-efficacy	-.59	.09					-	-.54***	-.43***	.27***	.13*	-.48***
7. Cognitive anxiety/Worry	.28	-.73						-	.65***	-.35***	-.20**	.66***
8. Somatic anxiety	.69	-.35							-	-.25***	-.05	.46***
9. Interpretation of anxiety	.71	.03								-	.20**	-.25***
10. Impact of message	.29	.86									-	-.10
11. Negative self-talk	.70	-.07										-

*Note*.

* = *p* < .05;

** = *p* < .01;

*** = *p* < .001.

## Discussion

The experience of anxiety when speaking in public is common and can be debilitating. Within the communication apprehension literature, sustained research attention has been targeted toward identifying and treating the causes of this form of social anxiety [8]. Guided by research on public speaking anxiety treatment effectiveness, and by cross-disciplinary knowledge about the nature of performance anxiety, we tested an inoculation message that was designed to not only alleviate individuals’ speech-related anxiety, but also to enable them to interpret any residual nerves in a more positive manner. In doing so, this investigation used established persuasion (i.e., inoculation theory) principles to explore the effects of an anxiety-alleviating message on participants’ cognitive and somatic anxiety, as well as their appraisals about the effects of anxiety for their performance capabilities. Relative to recipients of an information-only control message, participants who received the inoculation treatment reported lower task-related anxiety prior to their speech, along with lower retrospective ratings of in-task somatic anxiety. Inoculated participants also reported that the message they received contributed to them being less concerned about their anxiety.

On a practical level, this study presents a novel, standardized, and easy to disseminate method for alleviating a highly prevalent form of anxiety. Individuals who are highly anxious about public speaking display greater self-focused, negative attention during speaking, as well as poorer speaking performance [58]. From a more holistic perspective, high levels of public speaking anxiety have, in some instances, also been shown to accompany other mood and/or anxiety disorders [4], an aversion to group and dyadic interaction [59], and problems in employment, educational, and social situations [5]. The practical significance of successful public speaking anxiety treatments, therefore, is underscored by the potential to (at least partially) offset this adverse affective and behavioral profile.

It is important to also highlight, however, that public speaking anxiety treatments—including the present study—are typically successful only in reducing (and not completely eliminating) presenters’ anxiety. For that reason, we also sought to examine whether an inoculation message may hold additional promise for modifying recipients’ *interpretations* of their remaining nerves. When examining differences on our ‘anxiety interpretation’ outcome within a multivariate framework (i.e., when adjusting for multiple comparisons), we did not find evidence of a significant interpretational effect (i.e., the univariate p value for the between-condition difference test was .027). Nonetheless, the effect size (Cohen’s *d*, illustrated in Table 1) for this between-condition difference did demonstrate evidence of a small-to-medium-sized effect, offering some insight relating to—and encouragement for future research aimed at examining—the potential effectiveness of inoculation messages for anxiety reappraisal with non-clinical samples performing ‘real-world’ activities (i.e., that participants had prepared for over a period of weeks, and that was performed in front of a relatively large audience). In support, participants in the inoculation treatment condition—relative to those in the control condition—did report that the information they received (i.e., the inoculation, relative to control, message) made them significantly less ‘worried’ about any nerves that they carried into the speech.

An important practical conclusion emerging from these findings is that the relatively lower anxiety levels among inoculation participants occurred whilst including respondents’ personality traits as a covariate, and without weakening perceptions about the importance of the activity. It might have been argued that the ‘reassuring’ nature of the inoculation treatment could have reduced speech anxiety simply by acting to allay perceptions about the significance or importance of the activity. It is noteworthy, therefore, that those who received the inoculation (relative to control) message actually reported significantly greater task importance, a perception that typically accompanies heightened (not reduced) anxiety [60,61]. For that reason, it is noteworthy that the treatment effect was observed for anxiety levels (i.e., pre-speech task-related anxiety and in-task somatic anxiety) whilst accounting for task importance as a covariate in our analyses.

As well as considering issues of practical significance, it is important to reflect on the conceptual contribution of these findings. Most notably, this study emphasizes the potential applicability of inoculation theory. Inoculation messages have traditionally been used with the goal of fostering attitudinal protection (i.e., instrumental, value-based judgments), and have been demonstrated to be efficacious in conferring resistance with this target construct [34]. Recent research indicates, however, that inoculation techniques may also be effective for inducing resistance in the face of attacks/challenges to other psychosocial variables, including agentic perceptions such as self-efficacy [31]. These findings highlight that the ‘reach’ of inoculation may also extend to the protection of emotional states, and may provide the foundation for charting inoculation’s effectiveness in terms of affective dimensions of resistance (e.g., enjoyment, interest [28,32]).

The abovementioned findings were consistent with our a priori hypothesis; however, we did fail to detect between-group differences on some primary variables. Specifically, we observed no significant difference between inoculation and control participants’ pre-task self-efficacy. As would be expected according to principles of self-efficacy theory [62], higher pre-task self-efficacy perceptions were significantly and negatively correlated with all anxiety indices. However, the treatment did not induce stronger self-efficacy perceptions among inoculation (relative to control) participants. Similarly, although we observed effect sizes that indicated a small-to-moderate effect (and a significance value for cognitive anxiety that would have been interpretable were it not for our adjusted criterion), there were no significant differences between groups in terms of task-related cognitive anxiety/worry and negative self-talk. As such, although there may be merit in considering worry- and self-talk-related effects of inoculation treatments in future, out findings preclude any inferences relating to the effectiveness of inoculation on these differences. It is difficult to interpret these non-significant findings without relying on speculative (i.e., not data-driven) explanations. Nonetheless, it may be noteworthy that each of these variables is primarily cognitive (as opposed to affectively-laden) in nature, and so it may be possible that the treatment acted more powerfully on affective concepts. That being the case, it would be interesting in future to include additional counterargument-refutation pairings in treatments such as this that specifically target self-doubt and counterproductive self-talk.

It is also worth considering why the inoculation group failed to experience more threat than the control condition, as would be consistent with most inoculation research and the assumptions of the theory. Previous inoculation studies have provided evidence of resistance in the absence of significant differences in threat between the control and experimental group [63], pointing to other processes, besides threat and counterarguing, that are at work in inoculation-conferred resistance. Indeed, it is possible that, despite not being forewarned about the likely challenges they may face during their presentation, those in the control group were—due to their previous experience of speaking in public—still cognizant of the nerves and difficulties that accompany such a performance. Alternatively, although our single-item measure of threat tapped into participants’ awareness of impending challenge, it did not assess their perceived vulnerability to those challenges. This measurement approach was most efficient in terms of time demands, but in future it may be worthwhile to also measure individuals’ perceived vulnerability toward succumbing to the threat [28].

It is important to balance the novel aspects and contribution of this study against design limitations and opportunities for advancement of the work. For example, in light of time restraints it was necessary to utilize single-item assessments for some constructs (e.g., anxiety interpretation, pre-speech task-related anxiety). We observed associations between these measures and relevant correlates that were largely consistent with theory; however, in future it may be advisable to incorporate multi-item assessments where possible. In addition, we evaluated the efficacy of this treatment using a single performance scenario, and as a result we were unable to test some valuable inoculation processes. For example, inoculation scholars have demonstrated the potential for inoculation effects to persist over-time [64], and that treatments on a given issue may confer cross-protection for related issues/attacks [65]. These considerations encourage further research that examines (a) whether the acute anxiolytic effects we observed may be retained over time, and (b) if the public speaking treatment that we employed—or a modification thereof—might provide ‘umbrella’ protection against anxiety in other stress-inducing scenarios (e.g., test-taking, job interviews, sport competition).

On a separate issue, although our aim was to capture participants’ self-report anxiety levels, it would have been informative had we supplemented our respondent ratings with heart rate data, physiological markers [24,25], or audience perceptions [21], to examine both the internal and the overt behavioral signatures that accompany this form of anxiety [8]. Indeed, a more comprehensive approach such as this would enable future assessment to be more consistent with the longstanding notion that one’s emotional responses (such as anxiety) can be measured according to three systems—one’s affective reports (as we assessed in this study), one’s physiological signals, and one’s overt behavioral responses [66,67]. The nature of the group-based presentation format also has implications for future measurement considerations. Most notably, despite participants being randomly assigned to groups, it may be valuable in future—in cases where a group-based procedure is employed—to assess and control for relevant in-group communicative and social factors (e.g., perceptions of support, cohesion) that might shape presenters’ anxiety levels/interpretations.

It would also be valuable in future to compare the efficacy of different anxiety-focused inoculation treatments. To elaborate, two of the three counterargument-refutation pairings in our message were focused on reducing the perceived demand placed on participants (i.e., through material highlighting the illusion of transparency and spotlight effect), whereas the remaining pairing provided information that was focused toward enhancing respondents’ perceived coping resources (i.e., the reappraisal material). In future, it would be worthwhile to more specifically compare the effectiveness of ‘demand minimization’ inoculation messages, in which the aim is to alleviate anxiety, with ‘resource maximization’ messages, in which the aim is to help individuals reappraise and more effectively manage their anxiety [21].

In conclusion, this study demonstrates that inoculation theory might provide a novel approach for alleviating (aspects of) public speaking anxiety. Indeed, the standardized and easy-to-disseminate messaging principles within inoculation treatments might make this a particularly appealing approach given the widespread prevalence of public speaking anxiety. In addition to offering a novel treatment strategy, this study also drew from the anxiety reappraisal literature [25] to demonstrate that it may be possible to help participants *reframe* as well as *reduce* their apprehension about public speaking, and encourages future research that establishes the scope and lasting effects of anxiety-focused inoculation. Although public speaking is recognized as one of the foremost stress-inducing evaluative contexts, these findings may help inform the treatment of acute anxiety across work, education, sport, music, theatre, and social settings.

## Supporting Information

S1 File Inoculation treatment. (DOCX)

S2 File Raw data file. (SAV)
