# Supplementary material for: Re-Thinking Anxiety: Using Inoculation Messages to Reduce and Reinterpret Public Speaking Fears
Source: PLoS One. 2017 Jan 26;12(1):e0169972. doi: 10.1371/journal.pone.0169972 (PMC5268460; doi:10.1371/journal.pone.0169972)
Supplement: S1 File — (DOCX) [file pone.0169972.s001.docx]

**S1 File: Inoculation Treatment**

You are about to participate in a group-based oral presentation. This presentation will be assessed and will contribute 20% toward your grade. Your overall score for this assessment will be determined partly by the performance of your group and partly by the performance of yourself as an individual. You are expected to talk as a group and engage the audience for 30 minutes, and the audience will remain silent during this time. At the conclusion of your presentation, your group will facilitate a short tutorial discussion with your classmates. Good luck with all parts of the assessment.

Previously, we obtained data to indicate that many students become nervous prior to and during their presentation. As a consequence, we wanted to do something to help students manage their worries. Below (in bold) are some common negative thoughts that people have about presentations and public speaking. Following each of these negative thoughts, we’ve provided some material that describes why each negative thought actually has little foundation. We hope (and think) that this information will help you to manage your feelings prior to your speech.

**“Everyone will see that I’m nervous”**

- This is a common concern, and researchers call it the *illusion of transparency*. Scientific evidence has proven that it is misguided to think that everyone can pick up on your nerves or anxiety.
- Research has consistently shown that audience members are much less likely to perceive public speakers’ internal states (e.g., nervousness) than what the speakers think [1,2,3].
- No matter what you think, any intense feelings you might be experiencing (such as nerves) are displayed too subtly to be detected by others [3].
- Your nerves, if you have them, are not likely to be picked up by audience members – whether in this presentation, or in future talks you might give.

**“Everyone is judging me and my appearance during my talk”**

- This is another common thought, and researchers call it the *spotlight effect.* A lot of research indicates that people overestimate the extent to which others are actually attending to, and judging, their external appearance [4,5].
- This means that other people are not judging you and your appearance to the extent that you think they are.
- All of the audience members will present themselves at some point, and they are watching your presentation to learn about your group’s work; they’re not here with the goal of judging you.

**“I’m not going to do well in my speech because I’m nervous”**

- Researchers at some of the world’s best universities – including Yale [6] and Harvard [7] – have shown that the effects of stress and anxiety are largely dependent on *how the symptoms are interpreted*.
- For instance, anxiety and excitement share very similar physical symptoms; what seems to be important is how you *choose* to interpret the symptoms [7]. What this means is that anxiety, if you experience it, is not necessarily a bad thing at all, and might even help you feel ‘switched on’ and ready.
- In support, the correlation between anxiety and speech performance among presenters in this class last year was -0.16, which is a very small relationship. The people who were highly nervous didn’t do any worse in their presentation. Even if you feel anxious about your presentation, you don’t need to think of this as a threat to how well you’ll perform.

1. Behnke, R. R., Sawyer, C. R., & King, P. E. (1987). The communication of public speaking anxiety. *Communication Education, 36,* 138-141.
2. Mansell, W., & Clark, D. M. (1999). How do I appear to others? Social anxiety and processing of the observable self. *Behaviour Research and Therapy, 37,* 419-434.
3. Savitsky, K, & Gilovich, T. (2003). The illusion of transparency and the alleviation of speech anxiety. *Journal of Experimental Social Psychology,* *39,* 618-625.
4. Gilovich, T., Kruger, J., & Medvec, V. H. (2002). The spotlight effect revisited: Overestimating the manifest variability of our actions and appearance. *Journal of Experimental Social Psychology, 38,* 93-99.
5. Gilovich, T., Medvec, V. H., & Savitsky, K. (2000). The spotlight effect in social judgment: An egocentric bias in estimates of the salience of one’s own actions and appearance. *Journal of Personality and Social Psychology, 78,* 211-222.
6. Crum, A. J., Salovey, P., & Achor, S. (2013). Rethinking stress: The role of mindsets in determining the stress response. *Journal of Personality and Social Psychology, 104,* 716-733.
7. Brooks, A. W. (2014). Get excited: Reappraising pre-performance anxiety as excitement. *Journal of Experimental Psychology: General, 143*, 1144-1158.
